# Efgartigimod Combined With Steroid Treatment for HAM/TSP: A Case Report

**DOI:** 10.1002/acn3.70156

**Published:** 2025-07-30

**Authors:** Jiahui Zeng, Wotu Tian, Jingran Yang, Yanping Yang, Li Cao, Xinghua Luan

**Affiliations:** ^1^ Department of Neurology Shanghai Sixth People's Hospital Affiliated to Shanghai Jiao Tong University School of Medicine Shanghai China; ^2^ Shanghai Mental Health Center Shanghai Jiaotong University School of Medicine, Shanghai Intelligent Psychological Evaluation and Intervention Engineering Technology Research Center (20DZ2253800), Shanghai Key Laboratory of Psychotic Disorders Shanghai China; ^3^ Shanghai Neurological Rare Disease Biobank and Precision Diagnostic Technical Service Platform Shanghai China; ^4^ Department of Emergency Medicine Shanghai Sixth People's Hospital Affiliated to Shanghai Jiao Tong University School of Medicine Shanghai China

**Keywords:** corticosteroids, efgartigimod, HTLV‐1, immune modulation, tropical spastic paraparesis

## Abstract

HTLV‐1‐associated myelopathy/tropical spastic paraparesis (HAM/TSP) is a progressive neurological disorder with limited treatment options. We report a 54‐year‐old female with decade‐long, progressive HAM/TSP, previously refractory to rituximab, who experienced worsening spastic paraparesis and neurogenic bladder dysfunction. She showed remarkable improvement in spasticity, bladder function, and quality of life following combination therapy with efgartigimod and corticosteroids. This case highlights efgartigimod's potential as an adjunctive therapy for refractory HAM/TSP, suggesting a new immune modulation strategy and warranting further research into combination treatments.

## 1 Introduction

Human T‐lymphotropic virus 1 (HTLV‐1)‐associated myelopathy/tropical spastic paraparesis (HAM/TSP) is a progressive neurological disorder that remains endemic in specific regions, particularly in Japan, the Caribbean, Central Africa, and South America [1, 2]. Following prolonged latency periods of infection, HAM/TSP typically manifests in middle‐aged adults as a slowly progressive disorder characterized by lower extremity weakness, diffuse hyperreflexia, clonus, impaired vibration sense, and bladder dysfunction due to detrusor insufficiency, while cognitive function and upper extremity strength typically remain preserved [3]. While the complete pathogenic cascade is still being elucidated, HTLV‐1 primarily targets CD4+ T cells, with additional involvement of CD8+ T cells, B cells, and neural cells including astrocytes, ultimately leading to immune‐mediated damage to the spinal cord and chronic disability [4]. Diagnosis is established through detection of HTLV‐1 antibodies in blood and cerebrospinal fluid, supported by clinical manifestations and neuroimaging findings [1, 5].

Treatment of HAM/TSP presents a significant clinical challenge, with conventional immunosuppressive therapies often providing limited relief [6, 7]. Efgartigimod, a human IgG1‐derived Fc fragment that targets the neonatal Fc receptor (FcRn), represents a novel therapeutic approach. By blocking FcRn, efgartigimod accelerates the degradation of circulating IgG antibodies, potentially reducing pathogenic autoantibodies involved in neuroinflammatory conditions [8]. This mechanistic rationale is supported by recent evidence demonstrating its efficacy in treating generalized myasthenia gravis, where it produced rapid clinical improvement [9]. This case report documents our experience with a novel therapeutic approach in a female patient with HAM/TSP, combining prednisolone with efgartigimod. While long‐term low‐dose prednisolone has shown effectiveness in slowing HAM/TSP progression [10], some patients may still experience disease progression. Efgartigimod's targeted mechanism of FcRn blockade offers a potentially complementary approach to modulating pathogenic immune responses. This case highlights the feasibility of combining this novel immunomodulatory therapy with established corticosteroid treatment in HAM/TSP.

## 2 Case Presentation

This study was approved by the Ethics Committee of Shanghai Sixth People's Hospital Affiliated to Shanghai Jiao Tong University School of Medicine (Approval No. 2021‐219), and written informed consent was obtained from the patient. A 54‐year‐old Chinese woman presented with progressive difficulty in walking for over 10 years. The initial symptoms included inability to place her heels on the ground when standing and heel pain during initial steps. Her medical history was significant for receiving a blood transfusion during childbirth in her early twenties. Her condition gradually worsened with increasing lower limb spasticity, weakness, and urinary symptoms. In September 2019, she was diagnosed with HAM/TSP at Fujian Medical University First Affiliated Hospital based on positive HTLV‐1 antibodies in both serum and cerebrospinal fluid, with genetic testing ruling out spinocerebellar ataxia type 3 (SCA3) through normal CAG repeat analysis of the ATXN3 gene. Family history was negative for similar neurological symptoms, as evidenced by a detailed pedigree analysis. Between September 2019 and mid‐2022, the patient received four courses of rituximab therapy. This resulted in an initial decrease in her HTLV‐1 proviral load, from 11.03% in September 2019 to 9% in March 2020. However, these therapeutic benefits were not sustained. Over the 2 years following the completion of the rituximab course, the patient's condition gradually worsened, leading to severe neurogenic bladder and worsening spasticity by early 2024 and prompting her to seek treatment at our hospital. Given the progressive nature of her symptoms, combination therapy with corticosteroids and efgartigimod was initiated. The patient declined a repeat HTLV‐1 proviral load assessment upon admission.

Upon admission in November 2024, neurological examination revealed spastic paraparesis, increased muscle tone predominantly in the lower limbs, bilateral hyperreflexia (+++), and positive bilateral Babinski signs. A detailed muscle strength assessment using the MRC scale was performed, which showed weakness in the upper limbs (grade IV to IV+) and more pronounced weakness in the proximal lower limbs (hip and knee strength at grade IV+), while distal strength in the lower limbs was preserved (grade V). Laboratory tests confirmed persistent HTLV‐1 infection with positive proviral DNA and antibodies. A comprehensive immunological assessment upon admission revealed significant polysystemic immune dysregulation. Immunoglobulin analysis showed polyclonal hypergammaglobulinemia, with markedly elevated IgG (19.60 g/L) and IgA (5.85 g/L) levels. Consistent with this, both kappa and lambda serum free light chains were also elevated (5.60 g/L and 2.42 g/L, respectively), though immunofixation electrophoresis confirmed no monoclonal component was present. Flow cytometry analysis demonstrated a distorted lymphocyte subset distribution, including a reduced NK cell percentage (2.15%), an inverted CD4/CD8 ratio of 0.94, and an increased B cell percentage (16.36%). Furthermore, evidence of complement activation was noted, with elevated total complement activity (CH50 53 U/ML) and C1q levels (280 mg/L), while C3 and C4 levels remained within normal limits. MRI demonstrated diffuse spinal cord atrophy throughout the cervical and thoracic regions, along with mild disc protrusion at multiple levels of cervical and lumbar spine. Electromyography showed chronic neurogenic changes with increased motor unit potential amplitude and reduced recruitment in lower limbs, while nerve conduction studies were normal. Notably, admission labs also indicated a concurrent urinary tract infection (urinary white blood cells: 2401/μL; C‐reactive protein: 3.52 mg/L), which was treated accordingly. The urinary leukocyte count subsequently normalized by December 2, 2024.

**FIGURE 1:**
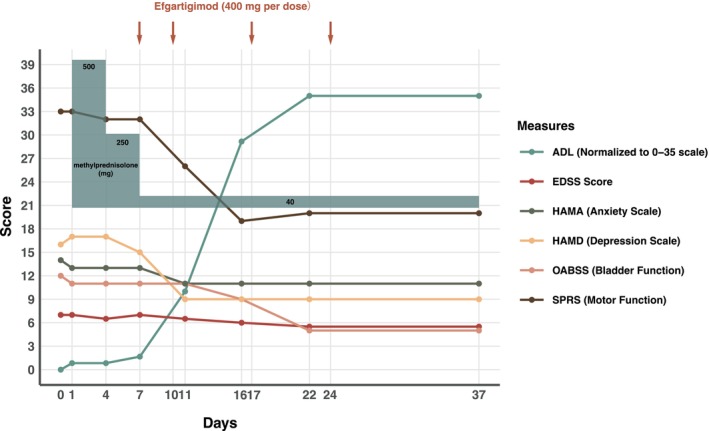
Clinical symptom changes following the combined use of corticosteroids and efgartigimod in treating HTLV‐1‐associated tropical spastic paraparesis (HAM/TSP). The patient received methylprednisolone pulse therapy (initial dose 500 mg, tapered to 250 mg, maintenance dose 40 mg), and four doses of efgartigimod (400 mg per dose). The graph shows changes in various clinical scores: ADL, Activities of Daily Living; Modified Barthel Index scores of 0–100, normalized to a 0–35 scale for visualization), EDSS, Expanded Disability Status Scale HAMA, Hamilton Anxiety Rating Scale; HAMD, Hamilton Depression Rating Scale; OABSS, Overactive Bladder Symptom Score; SPRS, Spasticity Rating Scale.

Given the progressive nature of her symptoms despite previous treatments, the patient received combination therapy with methylprednisolone pulse therapy (500 mg/day for 3 days, followed by 250 mg/day for 6 days, and subsequently tapered to a 40 mg oral prednisone maintenance dose) and efgartigimod (400 mg weekly for 4 weeks). The patient experienced facial flushing during the first 3 days of high‐dose methylprednisolone treatment, which gradually resolved over the subsequent 3 days. Treatment response was monitored using multiple validated scales (Figure 1). A measurable improvement in motor strength was noted in the upper limbs, which improved from MRC grade IV+ at admission to grade V. The Spastic Paraplegia Rating Scale (SPRS) score improved from 32 to 19, and EDSS decreased from 7.0 to 5.5; a detailed breakdown of the stepwise improvements in EDSS functional systems is provided in Table S1. The Modified Ashworth Scale (MAS) showed significant improvement in spasticity, particularly in elbow, wrist, and ankle movements; a detailed breakdown of these scores is provided in Table S2. Activities of daily living (ADL) score, as measured by the Modified Barthel Index [11], showed a score increase from 55 to 95, and quality of life showed marked improvement. The patient's urinary symptoms also improved substantially, with Overactive Bladder Symptom Score (OABSS) decreasing from 11 to 5. At 2‐month follow‐up, the patient maintained stable clinical improvement with no other significant adverse events.

## 3 Discussion

Efgartigimod, the first FDA‐approved neonatal FcRn antagonist for the treatment of gMG, has demonstrated significant efficacy [12]. In the ADAPT phase III trial, 44 out of 65 (67.7%) patients in the efgartigimod group achieved a response on the MG‐ADL scale, compared to only 19 out of 64 (29.7%) in the placebo group [9]. Efgartigimod works by blocking the interaction between FcRn and IgG, thereby accelerating IgG catabolism and reducing pathogenic autoantibody levels. This mechanism is similar to plasma exchange but is more selective, offers targeted IgG depletion, and provides a more sustainable treatment option [13]. HAM/TSP, an autoimmune disease, is characterized by chronic inflammation and immune activation due to HTLV‐1 infection, antibody‐mediated pathology, and the involvement of infected T cells [1, 14, 15]. While HAM/TSP is classically considered T‐cell‐mediated, our rationale for targeting the humoral system was based on evidence of its significant role in the underlying pathology. The role of humoral immunity in this context is nuanced; while some studies, such as Tang et al. [16], suggest a protective function for certain antibodies, other lines of evidence [17] support a pathogenic role, potentially through mechanisms like molecular mimicry and local production by activated B cells within the central nervous system. This complexity led us to hypothesize that the net effect of the humoral response is the critical factor and that, in our patient, the pathogenic burden far outweighed any neutralizing benefit. Supporting this hypothesis, our patient demonstrated marked polyclonal hypergammaglobulinemia (IgG 19.60 g/L) alongside clear evidence of complement activation (elevated CH50 and C1q). We reasoned that this dysregulated humoral state represented a valid therapeutic target and that efgartigimod would contribute to clinical improvement by reducing the overall IgG burden and disrupting the antibody‐driven inflammatory feedback loop. A review of therapeutic strategies explored for HAM/TSP over the past two decades (Table S3) reveals that FcRn inhibition has not yet been investigated in this context. However, it should be noted that most of these trials, except for mogamalizumab studies, have been small and lacked placebo controls, and there is currently no proven, consistently effective treatment for HAM/TSP.

The patient reported in this case had received four cycles of rituximab therapy over a 3‐year period between 2019 and 2022, along with concurrent treatments including baclofen, melatonin, mecobalamin, Rogaiquan, Kinadol, and idebenone. Although these treatments initially led to clinical symptom improvement and a fluctuating decline in viral load, with the patient still able to walk slowly and independently by 2022 (albeit with a fear of falling), the symptoms progressively worsened by 2024. Upon admission to our hospital, we implemented a novel combination therapy approach, distinct from the single‐agent interventions documented in Table S3. By combining efgartigimod's targeted reduction of pathogenic antibodies with the established anti‐inflammatory effects of corticosteroids, we observed comprehensive clinical improvements that surpassed the modest or symptom‐specific responses reported in previous studies. These improvements were evidenced by significant reductions across multiple measures: the SPRS score improved from 32 to 19, the EDSS score reduced from 7.0 to 5.5, and the ADL score increased from 55 to 95. Although the precise mechanism of HTLV‐1‐induced neurological damage remains unclear, our combined treatment strategy targets multiple pathogenic pathways, and such a comprehensive response suggests our strategy of targeting multiple pathogenic pathways is a potentially effective approach. Nevertheless, these results must be interpreted with caution in the context of two significant confounding factors. First, the patient's recovery was likely aided by the resolution of a concurrent urinary tract infection, although the magnitude of functional improvement far exceeded a simple return to her pre‐infection baseline. Second, the concurrent use of corticosteroids makes it difficult to ascertain the independent contribution of efgartigimod. Therefore, future studies, particularly those examining efgartigimod monotherapy, are warranted to validate these promising findings.

Our findings demonstrate the therapeutic potential of combining efgartigimod and corticosteroids in HAM/TSP treatment. The rapid and comprehensive improvement in functional scores suggests that simultaneously targeting FcRn‐mediated antibody recycling and inflammatory pathways may provide superior outcomes compared to monotherapy approaches. This aligns with emerging evidence supporting the role of targeted immune modulation in HTLV‐1‐associated neurological disorders, particularly given the complex pathogenesis involving both antibody‐mediated and inflammatory components [18]. The marked clinical response observed in our patient particularly highlights the advantage of addressing multiple disease mechanisms concurrently. While these results are encouraging, they should be interpreted with caution as they represent a single case report, and the concurrent steroid therapy limits our ability to determine efgartigimod's independent contribution. These findings lay the groundwork for future research, particularly prospective clinical trials needed to validate the efficacy of both combined therapy and efgartigimod monotherapy in a larger patient population and to establish the optimal timing of intervention.

This case report highlights the promising potential of combining efgartigimod with corticosteroids for the treatment of HAM/TSP, particularly in patients with refractory symptoms. The patient in this case, who had not responded adequately to prior treatments, experienced significant improvements in spasticity, bladder function, and overall quality of life after initiating this novel combination therapy. Efgartigimod, by targeting immune responses through FcRn inhibition, offers a unique mechanism of action that, when paired with corticosteroids, could effectively modulate the chronic inflammation and neuroimmune damage associated with HAM/TSP. This case suggests that efgartigimod may provide a complementary approach to traditional treatments, reducing reliance on prolonged corticosteroid use while enhancing therapeutic outcomes. While further studies are needed to validate these findings and optimize treatment protocols, the encouraging response in this patient underscores the potential of efgartigimod as a valuable therapeutic tool in managing this challenging and progressive condition.

## Author Contributions

Jiahui Zeng and Wotu Tian contributed equally to patient care, data acquisition, and drafting of the manuscript. Jingran Yang and Yanping Yang provided clinical data collection and interpretation. Li Cao and Xinghua Luan conceived the report, supervised the work, and critically revised the manuscript. All authors approved the final version.

## Conflicts of Interest

The authors declare no conflicts of interest.

## Supporting information


**Table S1.** Improvement in EDSS Functional Systems During Combination Therapy.
**Table S2.** Modified Ashworth Scale (MAS) Scores During Treatment.
**Table S3.** Summary (1–15) of Clinical Studies Investigating Different Therapeutic Approaches for HAM/TSP (2005–2024).

## Data Availability

The data that support the findings of this study are available from the corresponding author upon reasonable request.
