# Supplementary material for: Efgartigimod Combined With Steroid Treatment for HAM/TSP: A Case Report
Source: Ann Clin Transl Neurol. 2025 Jul 30;12(9):1937–41. doi: 10.1002/acn3.70156 (PMC12455869; doi:10.1002/acn3.70156)
Supplement: Supplementary file 1 — Table S1. Improvement in EDSS Functional Systems During Combination Therapy. Table S2. Modified Ashworth Scale (MAS) Scores During Treatment. Table S3. Summary (1–15) of Clinical Studies Investigating Different Therapeutic Approaches for HAM/TSP (2005–2024). [file ACN3-12-1937-s001.docx]

**Supplemental material**

**STable1. Improvement in EDSS Functional Systems During Combination Therapy**

| **Date**  **(2024)** | **Treatment status** | **Pyramidal** | **Cerebellar** | **Brainstem** | **Sensory** | **Bladder/**  **Bowel** | **Visual** | **Mental** | **Ambulation** | **EDSS Total Score** |
| --- | --- | --- | --- | --- | --- | --- | --- | --- | --- | --- |
| 11-27 | Steroid Pulse +  1st EFG | 3 | 5 | 0 | 0 | 4 | 0 | 1 | 6.5 | 7 |
| 12-01 | Post-1st EFG | 3 | 4 | 0 | 0 | 4 | 0 | 0 | 6.5 | 6.5 |
| 12-06 | Post-2nd EFG | 2 | 2 | 0 | 0 | 2 | 0 | 0 | 6 | 6 |
| 12-12 | Post-3rd EFG | 2 | 2 | 0 | 0 | 1 | 0 | 0 | 6 | 5.5 |
| 12-27 | Post-4th EFG | 2 | 2 | 0 | 0 | 1 | 0 | 0 | 6 | 5.5 |

**Note:** The table details the step-wise improvement in the patient's clinical status as measured by the Expanded Disability Status Scale (EDSS). Scores for the eight individual Functional Systems (e.g., Pyramidal, Cerebellar) and the EDSS Total Score were recorded at baseline and key intervals during the 4-week treatment cycle. *EDSS Total Score: According to standard EDSS guidelines, for scores of 5.0 and higher, the total score is primarily determined by ambulation (walking) ability.

**Abbreviations:** EDSS, Expanded Disability Status Scale; EFG, Efgartigimod.

**STable 2: Modified Ashworth Scale (MAS) Scores During Treatment**

| **Muscle Group** | **Side** | **Steroid Pulse +**  **1st EFG** | **Post-1st EFG** | **Post-2nd EFG** | **Post-3rd EFG** | **Post-4th EFG** |
| --- | --- | --- | --- | --- | --- | --- |
| Elbow Flexion | L/R | 1/1 | 0/1 | 0/1 | 0/0 | 0/0 |
| Elbow Extension | L/R | 1/1 | 0/1 | 0/1 | 0/0 | 0/0 |
| Wrist Flexion | L/R | 0/1 | 0/1 | 0/1 | 0/0 | 0/0 |
| Knee Flexion | L/R | 2/2 | 1/2 | 1/2 | 1/2 | 1/2 |
| Knee Extension | L/R | 2/2 | 2/1 | 1/1 | 1/1 | 1/1 |
| Ankle Dorsiflexion | L/R | 2/1 | 1/1 | 1/1 | 1/2 | 1/2 |
| Ankle Plantarflexion | L/R | 2/1 | 1/1 | 1/1 | 1/1 | 1/1 |

Note: The Modified Ashworth Scale (MAS) ranges from 0 (no increase in muscle tone) to 4 (affected part rigid), with intermediate grades of 1 (slight increase with catch and release), 1+ (slight increase with catch followed by minimal resistance), 2 (marked increase but easily moved), and 3 (considerable increase with difficult passive movement). EFG, efgartigimod; L, left; R, right.

**STable 3. Summary**^1-15^ **of Clinical Studies Investigating Different Therapeutic Approaches for HAM/TSP (2005-2024).**

| **Author** | **Study Design** | **Sample Size** | **Mean Age (years)** | **Sex** | **Treatment** | **Duration**  **(months)** | **Primary Outcomes** |
| --- | --- | --- | --- | --- | --- | --- | --- |
| Matsuzaki et al. （2005） | Prospective trial | 10 | 49.7 | M/F | LcS (fermented milk) | 1 | NK cell activity↑; Spasticity↓ |
| Croda et al. （2008） | Open cohort | 39 | 47 | M/F | Methylprednisolone (1g/day) | 26.4 | ISS improvement 24.5%↑ |
| Olindo et al. (2011) | Single-center trial | 19 | 53.4 | M/F | Valproic acid | 24 | No significant disability improvement |
| Rafatpanah et al.  (2012) | Clinical trial | 13 | 34.7 | M/F | IFN-α | 6 | Motor function↑; Viral load↓ |
| Hassan et al.  (2013) | Case report | 1 | 72 | F | Combivir | 5 | Walking ability↑ |
| Nakamura et al.  (2013) | Open-label trial | 24 | 60.1 | M/F | Prosultiamine (300mg/d) | 3 | Motor function↑; Viral load↓ |
| Cochereau et al. | Case reports | 2 | 56 | F | Alemtuzumab | 10.5 | Myopathy improvement↑ |
| Nakamura et al.  (2014) | Clinical trial | 12 | 64.5 | M/F | Pentosan polysulfate | 2 | Motor function↑ |
| Viana et al.  (2014) | Case report | 1 | 21 | F | Interferon beta-1a (3,000,000 IU) | 2.5 | Muscle strength↑; rinary symptoms↑; paresthesia↑ |
| Montalvá et al  (2015). | Case report | 1 | 40 | M | Cyclosporine | 15.5 | Clinical improvement↑ |
| Akahata et al.  (2019) | Phase I trial | 9 | 52.8 | M/F | Hu-Mikβ1 | 3.75 | No clinical efficacy |
| Akahata et al.  (2021) | Pilot study | 16 | 53.5 | M/F | Raltegravir | 15 | No significant PVL change |
| Nozuma et al.  (2023) | Phase 2 trial | 20 | 67.8 | M/F | L-Arginine (20g) | 1 | Walking speed (Day 14)↑ |
| Sato et al.  (2024) | Phase 3 RCT | 67 | 62.2 | M/F | Mogamulizumab | 6 | ↓PVL; No motor improvement |

Abbreviations: HAM/TSP = HTLV-1-associated myelopathy/tropical spastic paraparesis; LcS = Lactobacillus casei Shirota; IFN-α = Interferon-alpha; ISS = Inflammatory Spinal Cord Score; PVL = Proviral Load; RCT = Randomized Controlled Trial; M = Male; F = Female; ↑ = increase/improvement; ↓ = decrease/reduction

**Reference**

1. Viana GM, Silva MA, Souza VL, et al. Interferon beta-1a treatment in HTLV-1-associated myelopathy/tropical spastic paraparesis: a case report. *Rev Inst Med Trop Sao Paulo*. 2014;56(5):443-5. DOI:10.1590/s0036-46652014000500013

2. Sato T, Nagai M, Watanabe O, et al. Multicenter, randomized, double-blind, placebo-controlled phase 3 study of mogamulizumab with open-label extension study in a minimum number of patients with human T-cell leukemia virus type-1-associated myelopathy. *J Neurol*. 2024;271(6):3471-3485. DOI:10.1007/s00415-024-12239-x

3. Sánchez-Montalvá A, Salvador F, Caballero E, et al. Cyclosporine for the treatment of HLTV-1-induced HAM/TSP: an experience from a case report. *Medicine (Baltimore)*. 2015;94(1):e382. DOI:10.1097/md.0000000000000382

4. Rafatpanah H, Rezaee A, Etemadi MM, et al. The impact of interferon-alpha treatment on clinical and immunovirological aspects of HTLV-1-associated myelopathy in northeast of Iran. *J Neuroimmunol*. 2012;250(1-2):87-93. DOI:10.1016/j.jneuroim.2012.05.004

5. Olindo S, Belrose G, Gillet N, et al. Safety of long-term treatment of HAM/TSP patients with valproic acid. *Blood*. 2011;118(24):6306-9. DOI:10.1182/blood-2011-04-349910

6. Nozuma S, Matsuura E, Tashiro Y, et al. Efficacy of l-Arginine treatment in patients with HTLV-1-associated neurological disease. *Ann Clin Transl Neurol*. 2023;10(2):237-245. DOI:10.1002/acn3.51715

7. Nakamura T, Satoh K, Fukuda T, et al. Pentosan polysulfate treatment ameliorates motor function with increased serum soluble vascular cell adhesion molecule-1 in HTLV-1-associated neurologic disease. *J Neurovirol*. 2014;20(3):269-77. DOI:10.1007/s13365-014-0244-8

8. Nakamura T, Matsuo T, Fukuda T, et al. Efficacy of prosultiamine treatment in patients with human T lymphotropic virus type I-associated myelopathy/tropical spastic paraparesis: results from an open-label clinical trial. *BMC Med*. 2013;11:182. DOI:10.1186/1741-7015-11-182

9. Matsuzaki T, Saito M, Usuku K, et al. A prospective uncontrolled trial of fermented milk drink containing viable Lactobacillus casei strain Shirota in the treatment of HTLV-1 associated myelopathy/tropical spastic paraparesis. *J Neurol Sci*. 2005;237(1-2):75-81. DOI:10.1016/j.jns.2005.05.011

10. Hassan S, Amer S, Zervos M. Tropical spastic paraparesis treated with Combivir (lamivudine-zidovudine). *J Clin Neurosci*. 2013;20(5):759-60. DOI:10.1016/j.jocn.2012.05.048

11. Enose-Akahata Y, Oh U, Ohayon J, et al. Clinical trial of a humanized anti-IL-2/IL-15 receptor β chain in HAM/TSP. *Ann Clin Transl Neurol*. 2019;6(8):1383-1394. DOI:10.1002/acn3.50820

12. Enose-Akahata Y, Billioux BJ, Azodi S, et al. Clinical trial of raltegravir, an integrase inhibitor, in HAM/TSP. *Ann Clin Transl Neurol*. 2021;8(10):1970-1985. DOI:10.1002/acn3.51437

13. Croda MG, de Oliveira AC, Vergara MP, et al. Corticosteroid therapy in TSP/HAM patients: the results from a 10 years open cohort. *J Neurol Sci*. 2008;269(1-2):133-7. DOI:10.1016/j.jns.2008.01.004

14. Cochereau D, Georgin-Lavialle S, Maisonobe T, et al. Rationale and efficacy of CD52 targeting in HTLV-1-associated myositis. *Joint Bone Spine*. 2014;81(4):362-5. DOI:10.1016/j.jbspin.2014.01.019

15. Araya N, Takahashi K, Sato T, et al. Fucoidan therapy decreases the proviral load in patients with human T-lymphotropic virus type-1-associated neurological disease. *Antivir Ther*. 2011;16(1):89-98. DOI:10.3851/imp1699
